# All green sulfolane-based solvent enhanced electrical conductivity and rigidity of perovskite crystalline layer

**DOI:** 10.1038/s41598-023-36440-6

**Published:** 2023-06-08

**Authors:** Akarapitch Siripraparat, Pimolrat Mittanonsakul, Pimsuda Pansa-Ngat, Chaowaphat Seriwattanachai, Pisist Kumnorkaew, Anusit Kaewprajak, Pongsakorn Kanjanaboos, Pasit Pakawatpanurut

**Affiliations:** 1 Department of Chemistry, Faculty of Science, Mahidol University, Bangkok, 10400 Thailand; 2 Center of Excellence for Innovation in Chemistry (PERCH-CIC), Faculty of Science, Mahidol University, Bangkok, 10400 Thailand; 3 School of Materials Science and Innovation, Faculty of Science, Mahidol University, Bangkok, 10400 Thailand; 4 National Nanotechnology Center (NANOTEC), National Science and Technology Development Agency, Thailand Science Park, Khlong Luang District, Pathum Thani 12120 Thailand

**Keywords:** Chemistry, Energy science and technology, Materials science

## Abstract

Industrial commercialization of perovskite solar cells not only depends on sufficient device performance, but also requires complete elimination of hazardous solvents in the fabrication process to enable sustainable development of the technology. This work reports a new solvent system based on sulfolane, $\gamma$-butyrolactone (GBL), and acetic acid (AcOH) as a significantly greener alternative to common but more hazardous solvents. Interestingly, this solvent system not only resulted in densely-packed perovskite layer of bigger crystal size and better crystallinity, the grain boundaries were found to be more rigid and highly conductive to electrical current. The physical changes at the grain boundaries were due to the sulfolane-infused crystal interfaces, which were expected to facilitate better charge transfer and provide stronger barrier to moisture within the perovskite layer, yielding higher current density and longer performance of the device as a result. In fact, by using a mixed solvent system consisting of sulfolane, GBL, and AcOH in the volume ratio of 70.0:27.5:2.5, the device stability was better and the photovoltaic performance was statistically comparable with those prepared using DMSO-based solvent. Our report reflects unprecedented findings of enhanced electrical conductivity and rigidity of the perovskite layer simply by using an appropriate choice of the all-green solvent.

## Introduction

The inorganic-organic hybrid perovskite solar cell (PSC) has intrigued many researchers mostly due to its performance potential and promising outlook^1–3^. The rapid development of PSC in recent years also makes it possible to achieve even better photovoltaic performance, especially given the fact that many reported power conversion efficiency (PCE) values have consistently trailed behind the theoretical value of 25.2%^3^, leaving ample rooms for further achievement. A typical hybrid perovskite crystal structure can be characterized as $ABX_{3}$, which consists of cation (A site), such as CH$_{3}$NH$_{3}^{+}$, HC(NH$_{2}$)$_{2}^{+}$, Cs$^{+}$, and Rb$^{+}$. The inorganic framework $BX_{3}$ consists of halide elements (X site) and the metal (B site), such as Pb$^{+}$ and Sn$^{+}$^4^. The recently reported PCE has reached up to 25.2%^3^, a level that represents a significant advance especially when compared with other photovoltaics, such as CdTe, copper indium gallium selenide (CIGS), as well as dye-sensitized and organic solar cells^5^.

Equally important to the development of photovoltaic performance is the impact on the environment and human health of the preparation process. Reports of high performance often relied on the use of hazardous chemicals, such as carcinogenic *N*-methyl-2-pyrrolidone (NMP) and *N*,*N*-dimethylformamide (DMF) and highly skin-penetrating dimethyl sulfoxide (DMSO), for the preparation of perovskite^6–10^. However, even though the use of these solvents could warrant high device performance, their toxicity toward the environment and human health could cause serious issues, especially when the technology is implemented at large scale^10^. Therefore, to develop PSC technology in a sustainable manner, the issue over the choice of solvents needs to be addressed.

In an effort toward greener preparation, the mixed solvent consisting of polyethylene glycol and $\gamma$-butyrolactone (GBL) was used to prepare the PbBr$_{2}$ precursor solution. The resulting PSC showed a PCE of up to 8.11%^11^. (R)-(+)-Limonene and 2-methyltetrahydrofuran could also be used as less-toxic antisolvents in the preparation of high-quality perovskite layer in the inverted PSC device, with PCE of up to 17.84%^12^. Tian and coworkers also suggested n-butanol as a green antisolvent for preparing perovskite films in large-area devices, which demonstrated a PCE of 13.85%^13^. Yavari and coworkers used anisole as a green antisolvent in the preparation of PSCs, which yielded impressive PCE of up to 20.5%^14^. Nevertheless, despite many efforts so far, the device stability remains a challenge^14^, which could be attributed to inefficient dissolution of perovskite precursors in the preparation process^15^. As a result, complete elimination of hazadous solvents has yet to materialize, and these solvents still play a role to varying degree in the fabrication process to facilitate solubility of chemical precursors.

In this work, the issue of solubility of perovskite precursors, particularly PbI$_{2}$ for methylammonium lead(II) iodide perovskite, was tackled in order to determine alternative solvent system for the preparation of perovskite. Based on the Hansen solubility analysis in assessing the solubility of PbI$_{2}$^15^, sulfolane (1$\lambda ^{6}$-thiolane-1,1-dione or tetramethylene sulfone) emerged as a promising solvent that contains a sulfone functional group similar to DMSO^16^. In addition to very low skin penetration in comparison with other solvents^16^, sulfolane is a polar aprotic solvent with high miscibility, which makes it possible to be used in mixed solvents with various other compounds. The relatively high viscosity and boiling point of sulfolane would not only help reduce vapor pressure within the devices during normal operation, but also promote better occupational health and safety for future large-scale manufacturing. To formulate green solvent alternative based on sulfolane^17^, green additive such as acetic acid (AcOH) was also introduced to assist the solubility of PbI$_{2}$ by reducing the supersaturated concentration of the precursor and facilitating the formation of pre-nucleation cluster^18^. In addition, green component such as $\gamma$-butyrolactone (GBL) was also used in the solvent mix to improve the solubility of PbI$_{2}$ in accordance with the Hansen solubility concept^10,19,20^.

The results showed that a new mixed solvent system consisting of sulfolane, GBL, and AcOH could deliver a PCE that was statistically comparable to the control device fabricated using toxic DMSO-based solvent. Perhaps more importantly, the 10-days stability test under 48–50% relative humidity revealed that the device fabricated using the sulfolane-based solvent became more stable. Surprisingly, the results showed that a new mixed solvent system consisting of sulfolane, GBL, and AcOH could enhance electrical conductivity and rigidity of the perovskite layer, which in turn delivered cell performance that was statistically comparable to the control device fabricated using toxic DMSO-based solvent. The stability test under 48–50% relative humidity also revealed that the device fabricated using the sulfolane-based solvent was more stable at least for over 10 days of testing.

## Experimental

### Meterials

Transparent, conductive, fluorine-doped tin oxide (FTO) glass (surface resistivity: 7 $\Omega$/sq) substrates were purchased from Solaronix. For the synthesis of TiO$_{2}$ compact layer, titanium isopropoxide (TTIP) and titanium(IV) butoxide (Ti(OBu)$_{4}$) from Fluka Analytical were used. Ethanol (95%) from Sigma-Aldrich was used as the solvent for the synthesis. Titanium chloride (TiCl$_{4}$) was used for the surface treatment of the TiO$_{2}$ layer. For the perovskite solution, PbI$_{2}$ (99.99%), CH$_{3}$NH$_{3}$I (99.5%), 2,2$^\prime$,7,7$^\prime$-tetrakis (N, N-di-p-methoxyphenilamine)-9,9$^\prime$-spirobiflourene (spiro-OMeTAD, 99.5%), lithium bis-(trifluoromethanesulfonyl) imide (Li-TFSI, 99.9%), and 4-tert-butylpyridine (TBP, 96%) were purchased from Sigma-Aldrich. Titanium(IV) isopropoxide (99.999%), chlorobenzene (99.9%), *N*,*N*-dimethylformamide (DMF, 99.9%), dimethyl sulfoxide (DMSO, anhydrous 99.9%), $\gamma$-butyrolactone (GBL), sulfolane, acetic acid (AcOH, 99%), and acetonitrile (99.9%) were also obtained from Sigma Aldrich. For the metal evaporation, gold metal (99.99%) and silver metal (99.9%) were purchased from Kurt J. Lesker company.

### Synthesis of TiO$_{2}$ compact layer and TiCl$_{4}$ treatment

The sol–gel method was used to prepare the TiO$_{2}$ compact layer. First, TTIP was mixed with ethanol (95%) using the volume ratio 7.83:92.17. Then 60 $\upmu$L of concentrated HCl was slowly added into the solution. The solution was stirred overnight before being coated on an FTO glass using spin coating method. Finally, TiCl$_{4}$ solution was used to treat the surface of the TiO$_{2}$ layer.

### Device fabrication

The fluorine-doped tin oxide (FTO) glass was cleaned in 3 sequential steps (Alconox detergent, water, and isopropanol) and then dried using N$_{2}$ gas. The process of each step was conducted under high relative humidity of 48–52%. TTIP was coated onto the FTO glass as an electron transport layer (ETL) using spin coating at 2000 rpm for 30 s, which was followed by annealing at 550 $^\circ$C for 1 h and a cool-down to room temperature. TiCl$_{4}$ solution was used to treat the TiO$_{2}$ surface and air-dried on a hotplate at 500 $^\circ$C for 2 h. After the substrate cool down, a solution containing 1.5 mol L$^{-1}$ PbI$_{2}$ and 1.5 mol L$^{-1}$ CH$_{3}$NH$_{3}$I (or MAI) was coated on a substrate using two-step spin coating method at 1500 rpm for 7 s and then at 3500 rpm for 50 s, followed by annealing at 80 $^\circ$C for 20 min. The solvents used in this step were GBL:DMSO of volume ratio 70:30 (denoted as GBL:DMSO), GBL:sulfolane of volume ratio 70:30 (denoted as GBL:sulfolane), and GBL:sulfolane:AcOH of volume ratio 70:27.5:2.5 (denoted as GBL:sulfolane:AcOH), which had been optimized according to the Hansen solubility model as shown in Table S1. The optimized volume ratio of GBL (70):DMSO (30) reported in the previous work^20^ was used as the control sample. The spiro-OMeTAD was prepared by dissolving 72.3 mg of spiro-OMeTAD, 28.8 $\upmu$L of TBP, and 17.5 $\upmu$L of Li-TFSI solution (520 mg of Li-TFSI in 1 mL of acetonitrile) in 1 mL chlorobenzene. The solution was then spin-coated onto the perovskite layer using two sequential steps (at 1500 and 4000 rpm) for 50 s each to obtain a hole transport layer (HTL). In a final step, gold metal was deposited via thermal evaporation as a counter electrode. The device structure can be denoted as FTO/c-TiO$_{2}$/MAPbI$_{3}$/HTL/Au.

### Characterization

The current density–voltage (*J–V*) parameters of the measured devices were obtained by using an NREL calibrated Keithley Model 2400 under simulated AM 1.5G solar irradiation at 100 mW cm$^{-2}$. The forward scan was operated from − 0.1 to 1.1 V. The ultraviolet–visible spectroscopy (UV–Vis) spectra were measured using a UV–Vis spectrophotometer (Shimadzu UV-2600). The photoluminescence (PL) was performed by using Horiba Fluro Max 4, with excitation at 500 nm and emission from 600 to 900 nm. The device structure used for the PL measurement was FTO/perovskite. Atomic force microscopy (AFM) measurements were carried out by using Park NX-10 with Forta cantilever with 1.6 N m$^{-1}$ spring constant. The AFM setup was calibrated using a sapphire sample. The topography images were obtained at a speed of 10 $\upmu$m s$^{-1}$, and the force slopes were collected with 0.2 N m$^{-1}$ set point. Surface current mapping for *J*$_{sc}$ and *V*$_{oc}$ conditions were done by conductive contact mode with a platinum iridium5 (PtIr$_{5}$)-coated PPP-CONTSCPt cantilever that has a 0.2 N m$^{-1}$ spring constant and resonance frequency of 25 kHz. The scan speed was 2 $\upmu$m$^{-1}$ and the setpoint at 15 nN under 0.2 mW cm$^{-2}$ illumination of microscope light with the bias − 0.6 V for *V*$_{oc}$ current mapping and without bias for *J*$_{sc}$ current mapping. The X-ray diffraction (XRD) measurement was performed using a PANalytical Aris with a setting at 8 mA and 40 kV. The XPS results were obtained using a Thermo Scientific (UK) with an X-ray source of monochromated Al 150 W.

## Results and discussion

### Effect of the new solvent systems on perovskite structure

**Figure 1:**
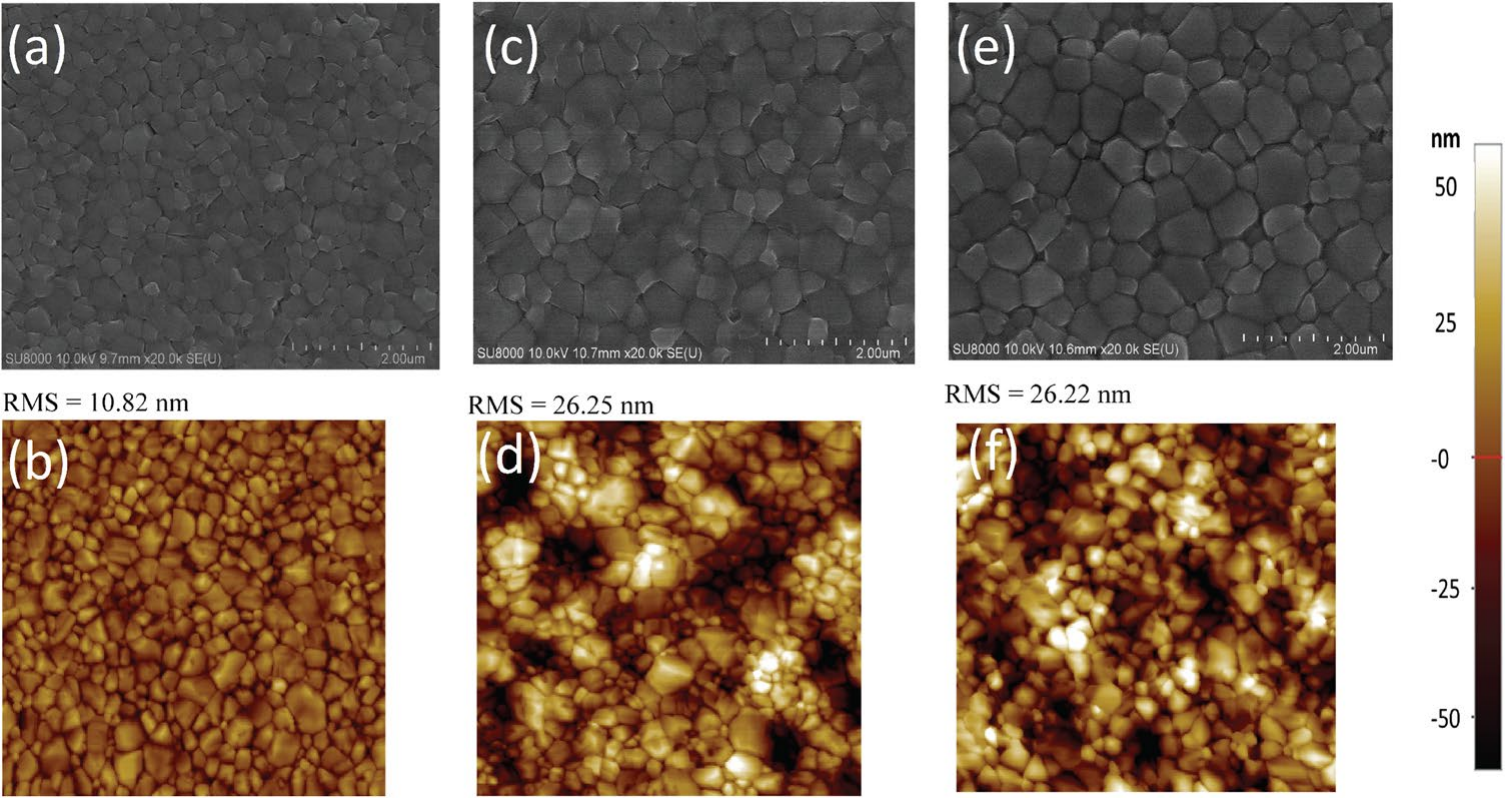
SEM and topological AFM images of $5.0 \times 5.0 \upmu$m in area of the perovskite films fabricated using (a and b) GBL:DMSO, (c and d) GBL:sulfolane, and (e and f) GBL:sulfolane:AcOH, respectively.

The top-view scanning electron microscopy (SEM) images of perovskite films fabricated using different solvents are shown in Fig. 1a,c,e. The results illustrated that, for perovskite film prepared using mixed solvent of GBL:sulfolane:AcOH, the crystals formed were more densely packed compared to the control sample prepared using GBL:DMSO. The perovskite crystals for the case of GBL:DMSO showed smaller sizes and higher amount of grain boundaries (Fig. 1a). This is in stark contrast to the formation of perovskite crystals using GBL:sulfolane and GBL:sulfolane:AcOH that showed larger crystal sizes of perovskite (Fig. 1c,e) resulting in increased surface roughness. The AFM topography images were consistent with the SEM images shown in Fig. 1b,d,f.

**Figure 2:**
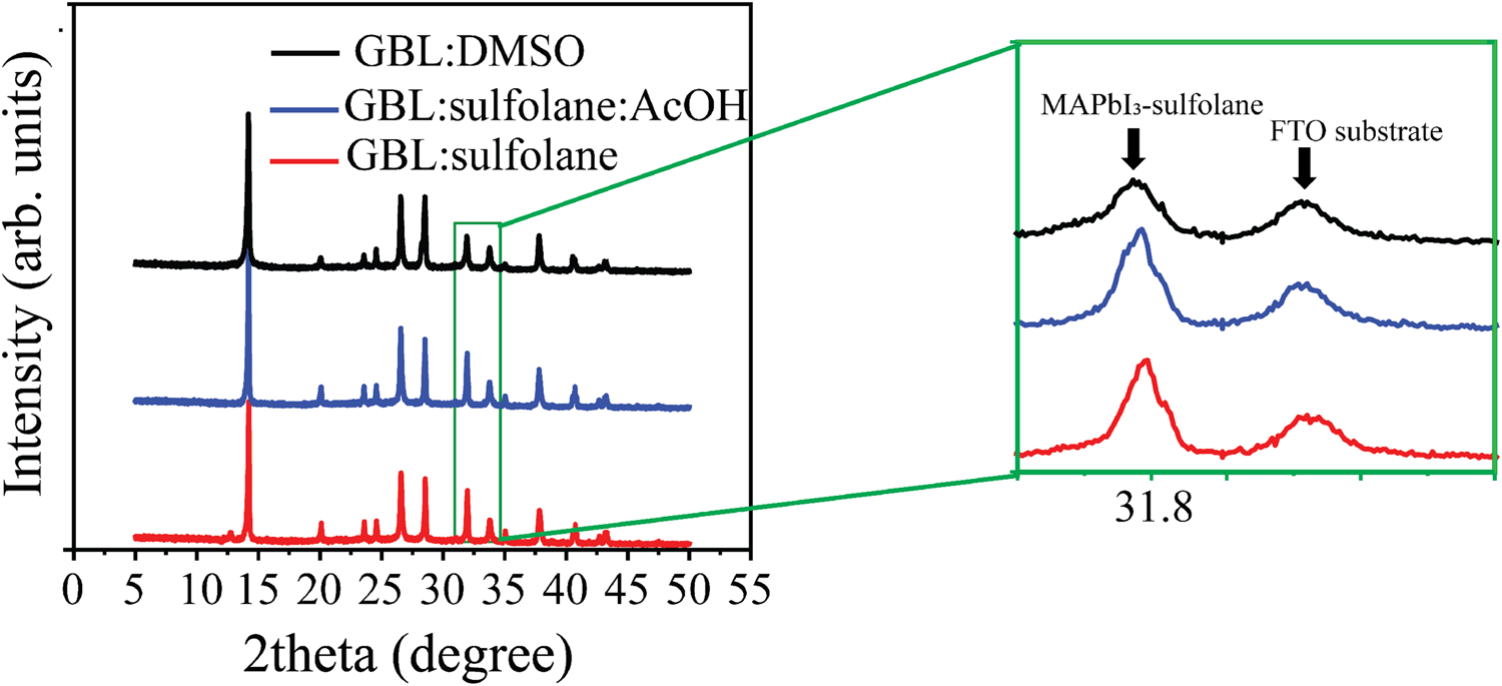
XRD patterns of the perovskite films prepared using different solvent systems. The zoom-in shows the peaks about 31.84$^\circ$, which corresponds to MAPbI$_{3}$-sulfolane phase^21^.

The quality of perovskite crystals was assessed by using XRD measurement on FTO/c-TiO$_{2}$/MAPbI$_{3}$. According to Fig. 2, the patterns of perovskite CH$_{3}$NH$_{3}$PbI$_{3}$ (or MAPbI$_{3}$) at 14.02$^\circ$, 28.4$^\circ$, and 31.84$^\circ$ can be indexed as (110), (220), and (310), respectively^22–25^. The XRD patterns, especially at the peak position of 31.84$^\circ$^24^, showed that the perovskite film prepared using a new solvent system of either GBL:sulfolane or GBL:sulfolane:AcOH revealed higher crystallinity when compared with the control sample prepared using GBL:DMSO. The improved crystallinity as a result of the sulfolane-based solvents, could be associated with the formation of the MAPbI$_{3}$-sulfolane close interaction^21^, while the structure of perovskite remained unchanged^26,27^. Furthermore, the crystal size calculated using the Scherrer’s equation for perovskite material prepared using GBL:sulfolane and GBL:sulfolane:AcOH was larger than those prepared using GBL:DMSO (see Table 1 and Fig. 1). The perovskite film prepared using GBL:sulfolane:AcOH showed no evidence of PbI$_{2}$ at 12.7$^\circ$, which indicated complete conversion of PbI$_{2}$ to perovskite^28–30^.

**Table 1:** Crystal size obtained using the Scherrer’s equation and *d*-spacing obtained using the Bragg’s law for perovskite materials prepared using GBL:DMSO, GBL:sulfolane, and GBL:sulfolane:AcOH.

Solvent system	2$\theta$ (degree)	FWHM	Crystal size (nm)	*d*-spacing (Å)
GBL:DMSO	14.28	0.0974	85.88	6.23
GBL:Sulfolane	14.22	0.0758	110.35	6.23
GBL:Sulfolane:AcOH	14.20	0.0870	96.59	6.23

**Figure 3:**
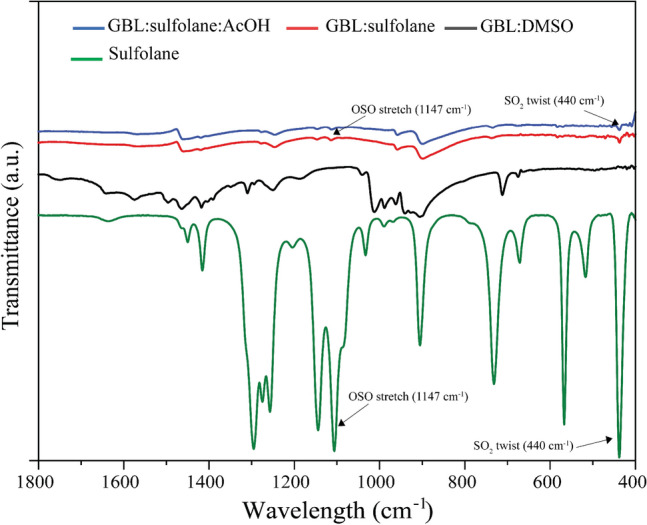
FTIR spectra of perovskite film prepared using different solvent systems.

**Figure 4:**
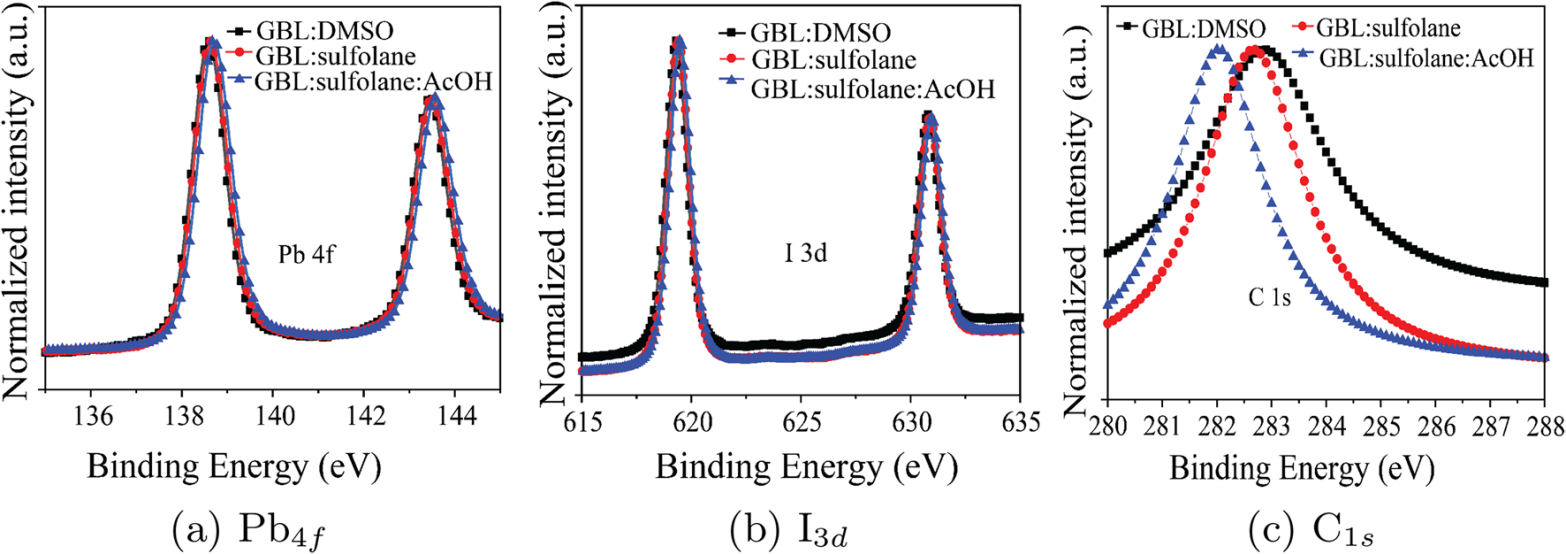
XPS results of perovskite prepared using different solvent systems.

It is interesting to note that the sulfolane-based solvents used in the perovskite preparation could play a role in the formation of perovskite layer. In fact, the FTIR peak positions of 440 and 1147 cm$^{-1}$ for the perovskite layer prepared using GBL:sulfolane and GBL:sulfolane:AcOH (Fig. 3) correspond to characteristic bonding of sulfolane in the perovskite films^31,32^ which also affirms the presence of MAPbI$_{3}$-sulfolane. The formation of MAPbI$_{3}$-sulfolane was further revealed by the XPS results. While most peaks of Pb$_{4f7/2}$, Pb$_{4f5/2}$^33^, I$_{3d}$ (Fig. 4a,b), as well as other peaks (Supplementary Fig. S1) were essentially similar among the perovskite prepared using different solvents, a significant red shift in C$_{1s}$ occurred with the case of GBL:sulfolane and GBL:sulfolane:AcOH (Fig. 4c). The lower binding energy observed for the case of sulfolane-based solvents could indicate strong solvation of the methylammonium cations (CH$_{3}$NH$_{3}^{+}$or MA$^{+}$) in perovskite by oxygen atoms in sulfonyl group^16^. Particularly in case of GBl:sulfolane:AcoH, interaction between MA$^{+}$ in perovskite and the carbonyl group of AcOH was possible^34^, resulting in chemical passivation^33,34^. Together with the XRD and IR spectra (Figs. 2 and 3), the presence of strong interaction between MAPbI$_{3}$ and sulfolane likely helped decelerate the crystallization process of perovskite^33^, which resulted in bigger perovskite crystals^21^.

In order to assess whether the presence of sulfolane and/or AcOH in the perovskite layer could translate into physical properties of the perovskite layer, mechanical pinpoint AFM was used to study the surface topography and mechanical properties of the layer. As revealed in Supplementary Fig. S2d–f, on average, the perovskite prepared using GBL:sulfolane and GBL:sulfolane:AcOH showed higher modulus than that of the perovskite film prepared using GBL:DMSO. Similarly, the deformation of perovskite films prepared using sulfolane-based solvents was lower than that of the sample prepared in DMSO-based solvent (Supplementary Fig. S3d–f). The increased rigidity of the perovskite layer when prepared using sulfolane-based solvents could be attributed to the presence of sulfolane, possibly in the areas of grain boundaries. Such an increased in rigidity may also help impede the penetration of moisture into the grain boundaries of the perovskite film, which could lead to better stability of the device^35^. These results were also consistent with the adhesion mapping shown in Supplementary Fig. S3a–c^35,36^.

**Figure 5:**
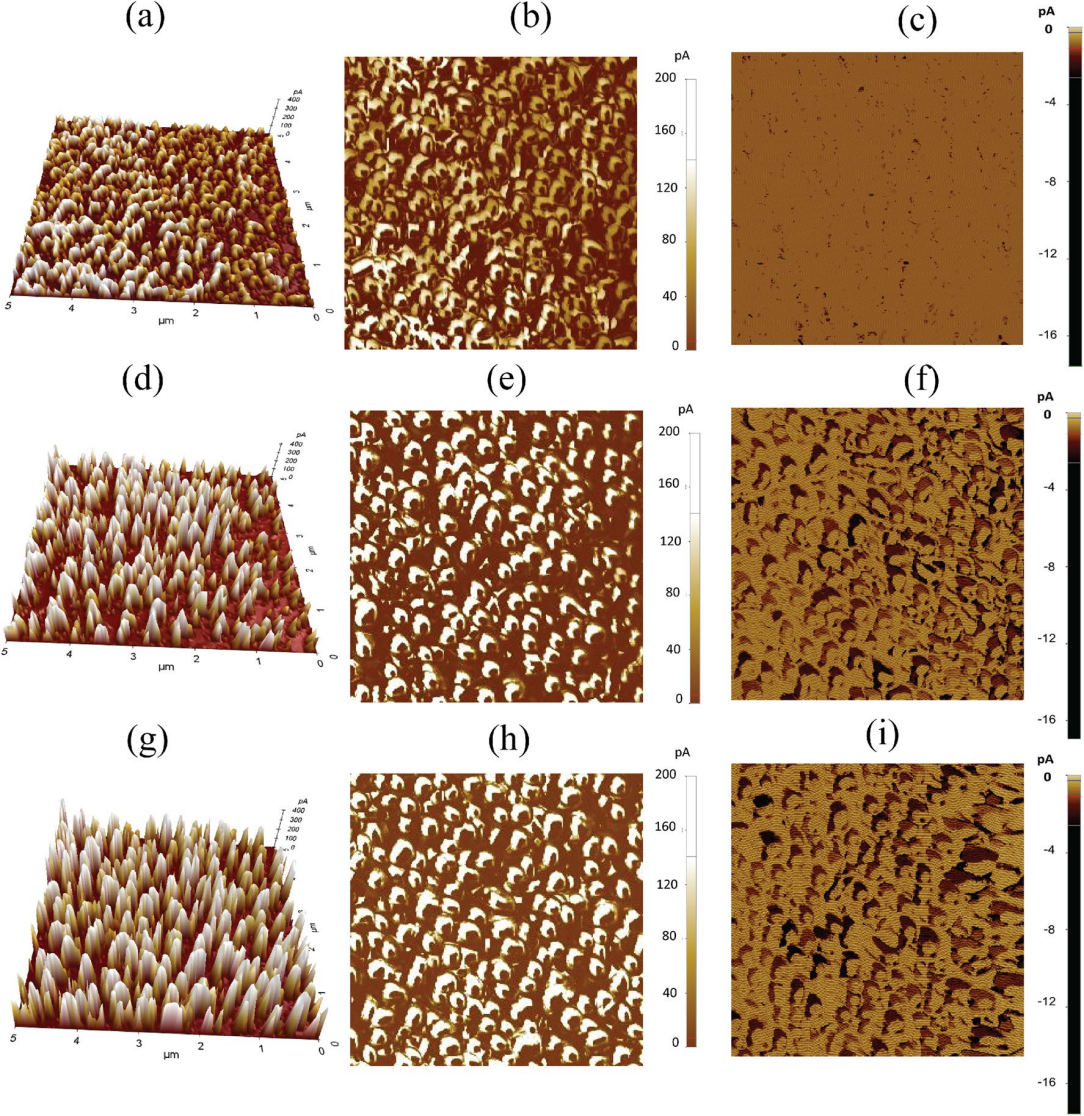
c-AFM (3D) and top surface current mapping (2D) at 0 V and at bias − 0.6 V (*V*$_{oc}$ mapping) of the perovskite films fabricated using (**a**–**c**) GBL:DMSO, (**d**–**f**) GBL:sulfolane, and (g-i) GBL:sulfolane:AcOH, respectively.

To probe the electronic properties of the surface of perovskite, conductive atomic force microscopy (c-AFM) was performed to obtain current mapping with no external bias under the illumination of microscope light with power 0.2 mW cm$^{-2}$. According to the results shown in Fig. 5d,e,g,h, the perovskite layers prepared using GBL:sulfolane and GBL:sulfolane:AcOH revealed average current of 75 pA and 96 pA, respectively, which were significantly higher than 33 pA of the sample prepared using GBL:DMSO (Fig. 5a,b). The improved charge transfer on the conductive surface when sulfolane-based solvents were used to fabricate the perovskite layer was likely originated from the presence of electron-rich, high-dipole moment sulfolane molecules^37,38^, at the grain boundaries of the perovskite^39^. This is consistent with the surface electrical current profiles shown in Supplementary Fig. S4d–f. The higher current density indicated enhancement in conductivity, especially with the solvent GBL:sulfolane:AcOH (Fig. 5g,h), which could facilitate better carrier separation and improve the short-circuit current density (*J*$_{sc}$) of the device^33^.

To further investigate electrical properties of the perovskite layer, *V*$_{oc}$ mapping (current maps under reverse bias) was measured in which a reverse bias of − 0.6 V was used to block the current generated at the normal band from hole blocking ability of electron transport layer and observe the current at the position of lower *V*$_{oc}$ area with high density of trap sites. The perovskite layers prepared using GBL:sulfolane and GBL:sulfolane:AcOH showed higher current leakage (Fig. 5f,i) when compared with the perovskite prepared using GBL:DMSO (Fig. 5c). The degree of current leakage indicates the degree of trap recombination sites at the surface of perovskite^40^. It is interesting to also note that, although the grain boundaries of perovskite prepared with sulfolane-based solvents showed favorable electrical current as revealed by c-AFM, the presence of trap recombination was rather significant and still posed an issue for device performance.

### Effect of the solvent on photoluminescence and absorption

**Figure 6:**
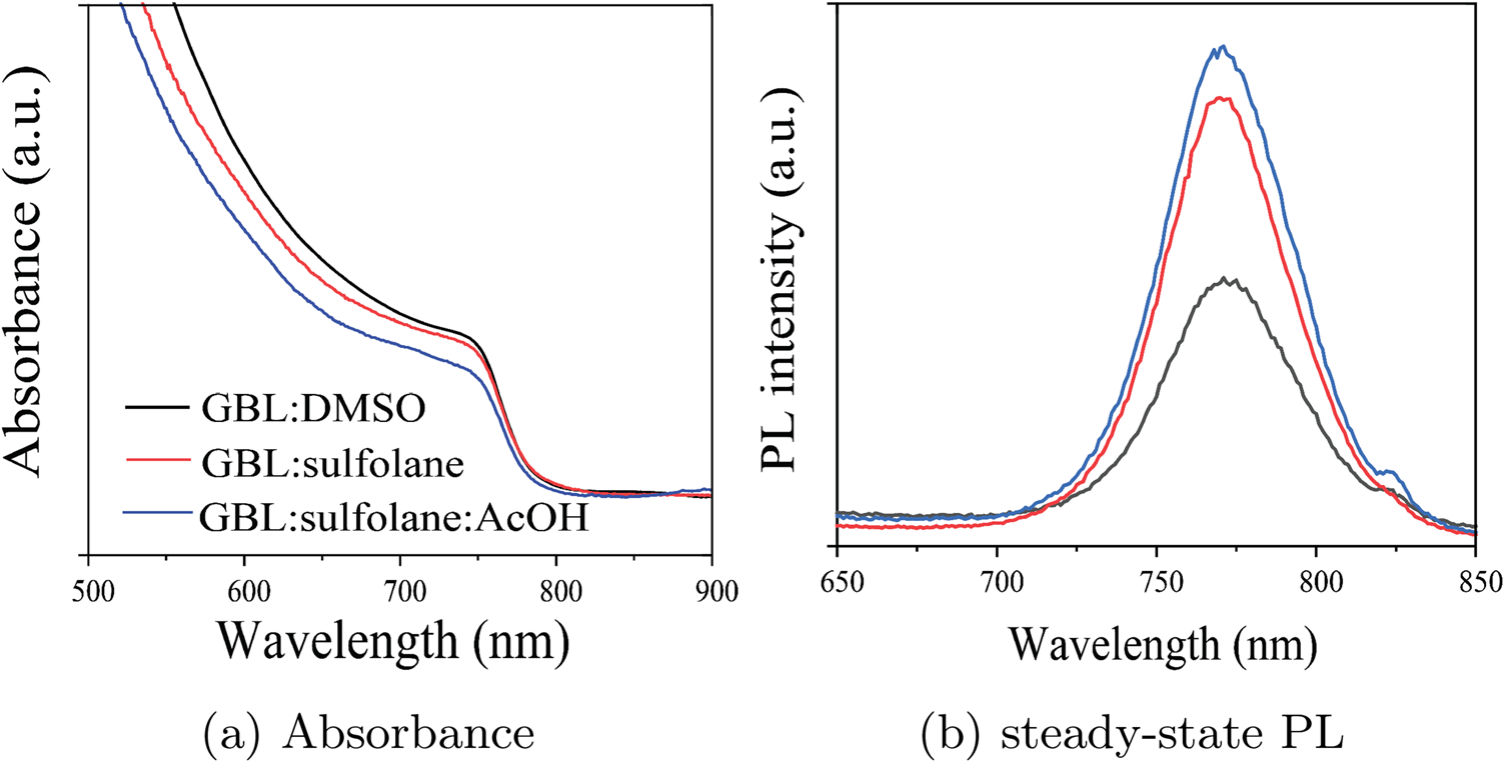
Light properties of perovskite films prepared using different solvent systems.

As shown in the UV–Vis spectra in Fig. 6a, minor differences in absorption intensity were observed at visible wavelengths among the perovskite samples prepared using different solvent systems. The onset of perovskite absorption remained essentially the same at 765 nm for all solvent conditions. However, the steady-state photoluminescence (PL) revealed significant differences when different solvents were used for the preparation of the device structure FTO/MAPbI$_{3}$. According to Fig. 6b, using GBL:sulfolane or GBL:sulfolane:AcOH showed higher PL intensity when compared to the case of GBL:DMSO. This result suggests that the perovskite prepared using sulfolane-based solvents likely showed higher radiative emission^11,41^, which is consistent with the cAFM results (Fig. 5g–i,), as well as higher current density (Table 2). We noted that all perovskite layers prepared using different solvent systems did not significantly differ in terms of thickness (see Supplementary Fig. S5).

**Table 2:** Photovoltaic parameters of PSCs prepared using GBL:DMSO, GBL:sulfolane, and GBL:sulfolane:AcOH.

Solvent system	PCE (%)$^{{a}}$	*FF*$^{{b}}$	*V*$_{oc}$ (V)$^{c}$	*J*$_{sc}$ (mA cm$^{-2}$)$^{d}$
GBL:DMSO	$14.90\pm 1.94$	66.40	0.98	20.15
GBL:Sulfolane	$12.86\pm 1.21$	57.48	0.93	23.43
GBL:Sulfolane:AcOH	$13.60\pm 1.45$	52.50	0.97	24.98

$^{a}$Power conversion efficiency; $^{b}$fill factor; $^{c}$Open-circuit voltage; $^{d}$ Short-circuit current density.

To assess the relative influence between the electron-hole and trap recombination, the time-resolved photoluminescence (TRPL) was also performed and analyzed via bi-exponential fit^42^.

According to Supplementary Table S2 and Fig. S6, the perovskite prepared using sulfolane-based solvents showed higher fraction of short-time component ($\alpha$2), which can be attributed to trap recombination at the perovskite interfaces^41^-consistent with the *V*$_{oc}$ mapping in Fig. 5c,f,i. Although the sulfolane-based solvent resulted in more trap sites, the average carrier lifetime was not significantly different from that of the perovskite prepared with GBL:DMSO (Supplementary Table S2). This could be due to the large crystal size of perovskite, especially in the presence of acetic acid in the solvent system, which prolonged the electron-hole recombination time component and helped recover *V*$_{oc}$ loss due to trap sites^33^.

### Effect of the solvent on performance and long-term stability

**Figure 7:**
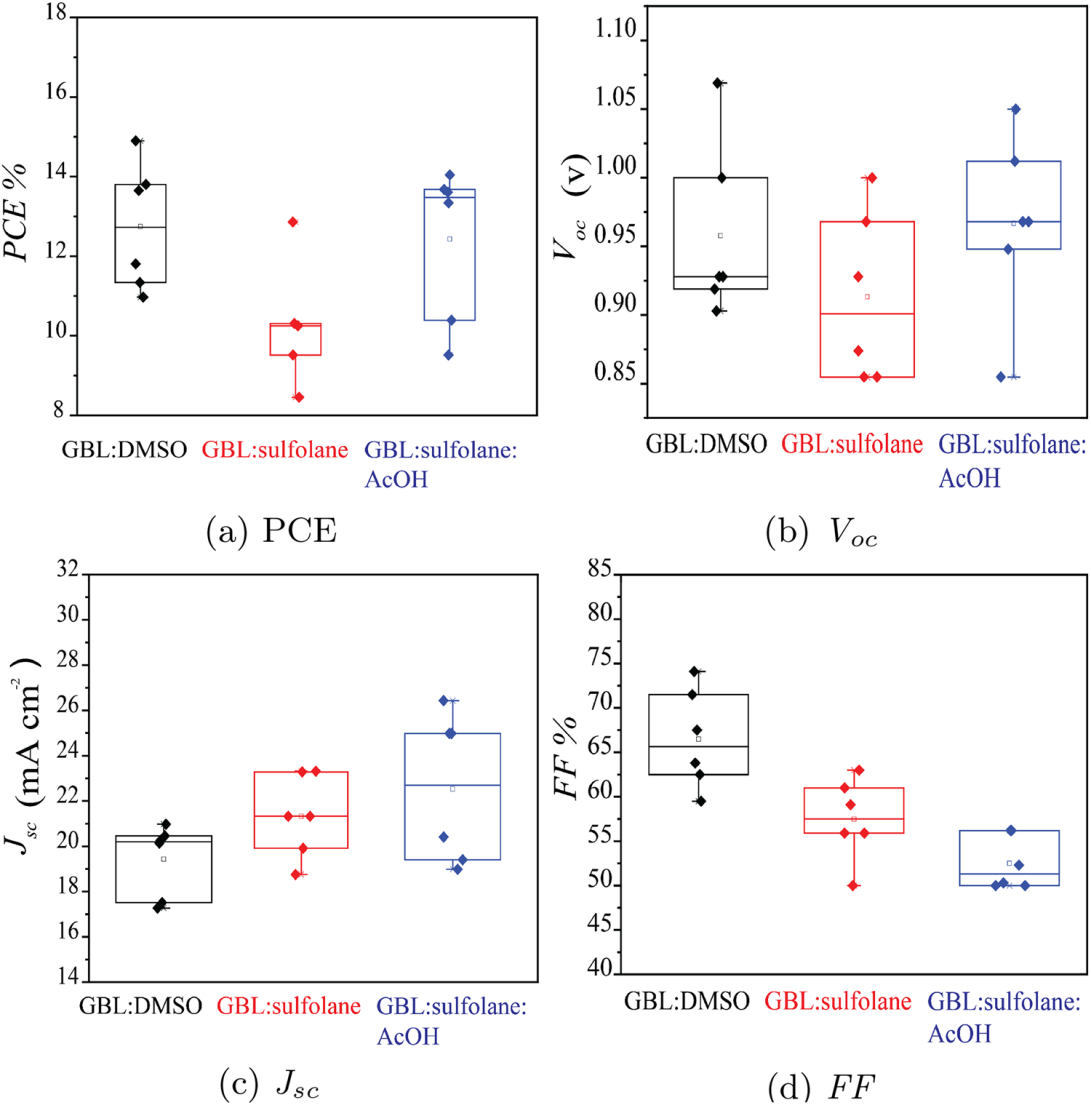
Device performance for perovskite prepared using different solvent systems.

According to the analysis of the *J–V* results (See Supplementary Fig. S7), the efficiency of the devices fabricated using GBL:sulfolane:AcOH was $13.60\pm 1.45$%, which was statistically similar to the value of $14.90 \pm 1.94$% for the control devices prepared using GBL:DMSO. As reported in Table 2 and shown in Fig. 7a,b, *J*$_{sc}$ of the devices fabricated using GBL:sulfolane:AcOH was higher in value compared with the control one prepared using GBL:DMSO. The enhanced *J*$_{sc}$ was likely due to higher crystallinity and conductivity of perovskite crystals, as suggested in Figs. 2 and 6b. Although the use of sulfolane-based solvents inadvertently increased the trap sites that affected *V*$_{oc}$, the chemical passivation of acetic acid in GBL:sulfolane:AcOH helped recover the *V*$_{oc}$ loss^33^. As a result, the *V*$_{oc}$ of device prepared with GBL:sulfolane:AcOH was essentially unchanged compared to the case of DMSO based solvent (Table 2). The fill factor (*FF*) of the devices fabricated using sulfolane-based solvents was, however, lower than the case of DMSO-based solvent (Fig. 7c). This could be attributed to the surface roughness of perovskite crystals prepared using sulfolane-based solvents (see Fig. 1), possibly preventing close interfaces between perovskite and adjacent layers^22^.

**Figure 8:**
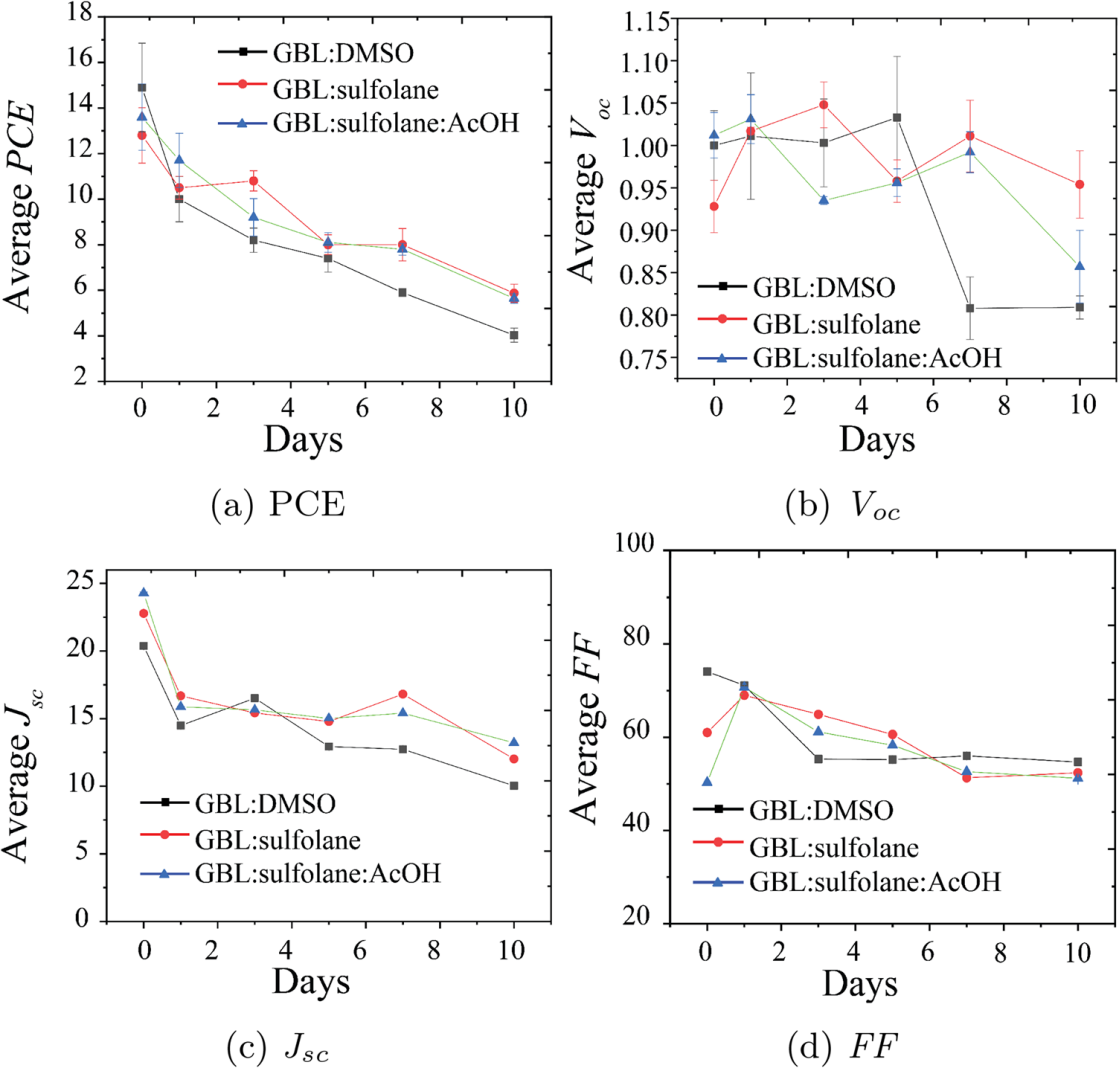
Average device performance during a period of 10 days under 48–52% RH for encapsulated PSC devices prepared using different solvent systems.

The use of sulfolane-based solvents not only yielded the performance on par with DMSO-based solvent, but also resulted in greater device stability over at least 10 days of testing. According to Fig. 8, the PSC devices prepared using GBL:sulfolane and GBL:sulfolane:AcOH revealed good stability. The unencapsulated devices prepared using GBL:sulfolane and GBL:sulfolane:AcOH showed about 5% drop in PCE under relative humidity (RH) of 48–50% (Fig. 8a). *J*$_{sc}$ and *V*$_{oc}$ also showed favorable trend for sulfolane-based solvents, revealing drops of about 8 mA cm$^{-2}$ and 0.01 V in *J*$_{sc}$ and *V*$_{oc}$, respectively (Fig. 8b,c). On the contrary, the control sample prepared using GBL:DMSO showed rapid deterioration in PCE, *J*$_{sc}$, and *V*$_{oc}$ by 9%, 10 mA cm$^{-2}$, and 0.02 V, respectively. Such a rapid decrease in performance was likely due to the smaller grain size with no passivation of sulfolane in perovskite^15,22^. Overall, these results indicated that the use of eco-friendly sulfolane-based solvents could improve both the device efficiency and the long-term performance. The use of acetic acid in the solvents led to more uniform crystals of perovskite and also helped eliminating unreacted PbI$_{2}$ that would have resulted in lower stability and efficiency of the devices^15,31^.

## Conclusion

The optimal condition for the all green solvent based on nontoxic sulfolane, GBL, and AcOH for the preparation of perovskite was reported. The perovskite crystals prepared using GBL:sulfolane and GBL:sulfolane:AcOH showed larger crystal sizes with higher rigidity. In contrast, the perovskite crystals prepared using GBL:DMSO were loosely packed with more grain boundaries. The XRD pattern also confirmed high crystallinity and preferential orientation of perovskite crystals when prepared using the green solvents reported. The steady-state PL also revealed higher radiative emission when sulfolane-based solvents were used in the preparation. With the presence of sulfolane remaining at the grain boundaries, the cAFM results indicated a significantly improved electrical conductivity was improved. Even though sulfolane-infused grain boundaries inadvertently promoted trap sites, strong interaction between perovskite and sulfolane, as well as acetic acid passivation, helped counter any loss in performance. In terms of the photovoltaic performance, PCE of $13.6 \pm 1.45$%, which was statistically comparable to $14.9 \pm 1.94$% of the control sample, was observed when the sulfolane-based solvents were used. Such a satisfactory conversion efficiency can be attributed to relatively high *J*$_{sc}$, afforded by improved crystallinity and conductive surface of perovskite.

When observed over a period of 10 days under high relative humidity of 48–52% without device encapsulation, the PSC devices prepared using GBL:sulfolane and GBL:sulfolane:AcOH showed stability superior to the case of DMSO-based solvent. Thus, the sulfolane-based solvents not only could yield better overall performance of the devices, but also offer environmentally conscientious approach toward present and future development of PSC. As large-scale PSC production becomes progressively feasible, these green alternative solvents could play an increasingly important role in protecting human health and minimizing detrimental impact on the environment.

## Supplementary Information

Supplementary Information.

## Data Availability

The datasets used and/or analyzed during the current study are available from the corresponding author on reasonable request.
