# Supplementary material for: All green sulfolane-based solvent enhanced electrical conductivity and rigidity of perovskite crystalline layer
Source: Sci Rep. 2023 Jun 8;13:9335. doi: 10.1038/s41598-023-36440-6 (PMC10250537; doi:10.1038/s41598-023-36440-6)
Supplement: Supplementary file 1 — Supplementary Information. [file 41598_2023_36440_MOESM1_ESM.pdf]

All Green Sulfolane-Based Solvent Enhanced Electrical Conductivity and Rigidity of Perovskite Crystalline Layer

Akarapitch Siripraparat,[†,‡,‖] Pimolrat Mittanonsakul,[†,‖] Pimsuda Pansa-Ngat,[¶] Chaowaphat Seriwattanachai,[¶] Pisist Kumnorkaew,[§] Anusit Kaewprajak,[§] Pongsakorn Kanjanaboos,[‡,¶] and Pasit Pakawatpanurut[*,†,‡]

†*Department of Chemistry, Faculty of Science, Mahidol University, Bangkok 10400, Thailand*

‡*Center of Excellence for Innovation in Chemistry (PERCH-CIC), Faculty of Science,Mahidol University, Bangkok 10400, Thailand*

¶*School of Materials Science and Innovation, Faculty of Science, Mahidol University, Bangkok 10400, Thailand*

§*National Nanotechnology Center (NANOTEC), National Science and Technology Development Agency, Thailand Science Park, Pathum Thani 12120, Thailand*

‖*Contributed equally to this work*

E-mail: pasit.pk@gmail.com

Additional Experimantal Data

Hansen Solubility

The Hansen theory is based on the idea of like-dissolve-like. The solubility between any pair of solvents is assessed according to the similarity of their interaction. The distance RA in this case is used to indicate the similarity of the Hansen solubility parameters (HSPs) between solute (δ_{d1}, δ_{p1}, δ_{h1}) and solvent (δ_{d2}, δ_{p2}, δ_{h2}), where δ_d is dispersion force, δ_p is polarity force, and δ_h is hydrogen-bonding force. RA can be calculated using equation 1.

$$[H]\mathrm{R}_A^2 = 4(\delta_{d2} - \delta_{d1})^2 + (\delta_{p2} - \delta_{p1})^2 + (\delta_{h2} - \delta_{h1})^2 \quad (1)$$

For a mixed solvent, the Hansen solubility parameter is calculated according to equation 2.

$$[H]\delta_2 = \sum A_n \delta_n \quad (2)$$

where A$_n$ and δ_n are the volume fraction and HSPs for each component, respectively.

Table S1: Different compositions of the solvents and the corresponding Hansen solubility parameters.

Volume ratio of solvent system (%)				Hansen solubility parameter			
GBL	DMSO	Sulfolane	AcOH	δ_d	δ_p	δ_h	R_A
70	30	0	0	18.12	16.54	8.24	7.93
70	0	30	0	18.00	17.02	8.15	7.70
70	0	27.5	2.5	18.08	16.93	8.10	7.80
70	0	25	5	18.17	16.85	8.06	7.90

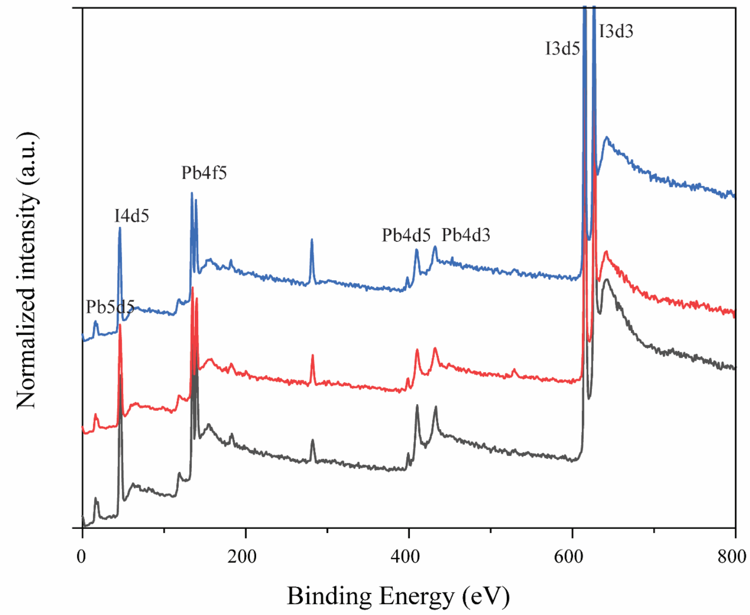

Figure S1: XPS spectra of the perovskite films prepared using different solvents, which indicate negligible change in perovskite compositions.

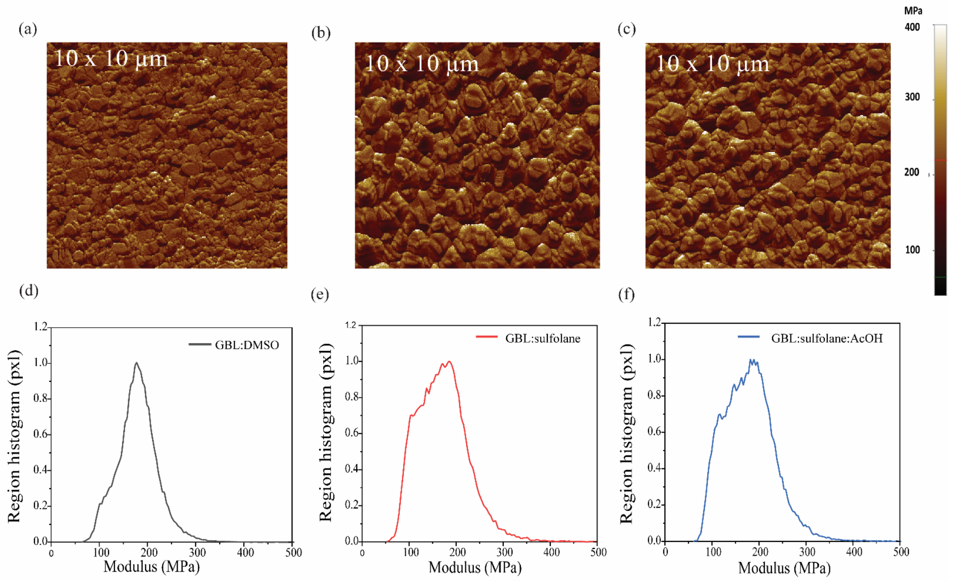

Figure S2: Topological AFM images and region histogram of Young's modulus of the perovskite films fabricated using (a-d) GBL:DMSO, (b-e) GBL:sulfolane, and (c-f) GBL:sulfolane:AcOH, respectively.

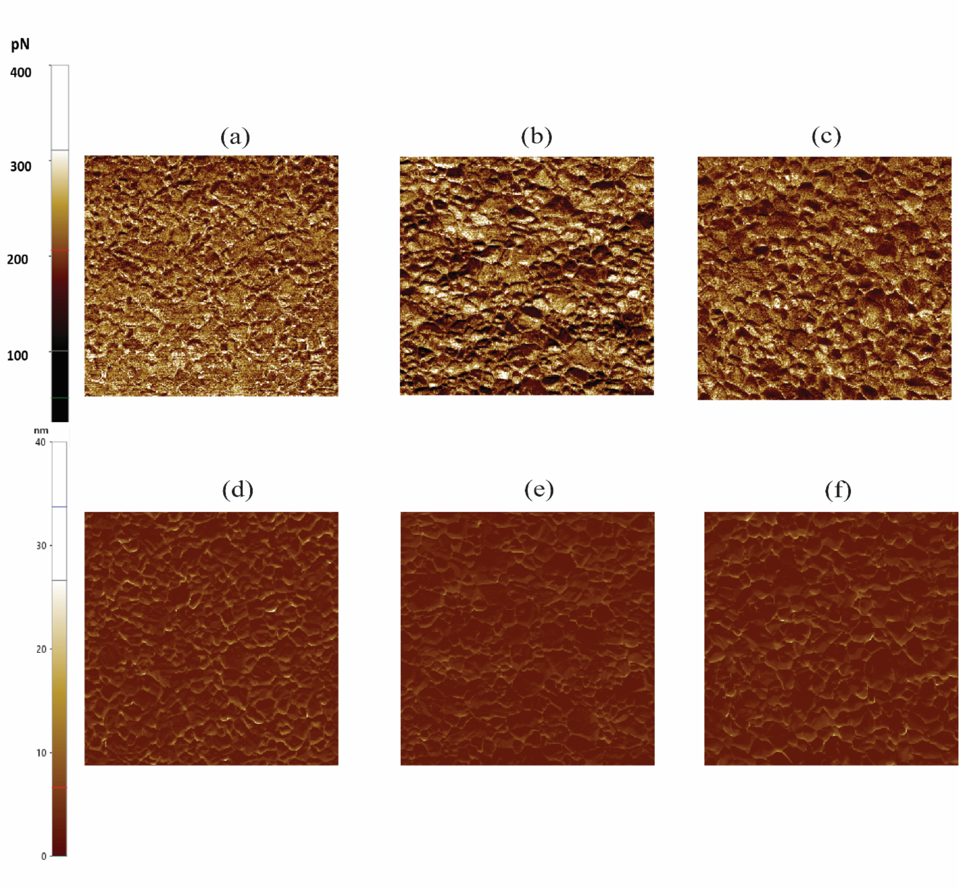

Figure S3: Atomic force microscopy (AFM) (a-c) adhesion mapping and (d-f) deformation mapping for the perovskite films fabricated using different solvent systems: GBL:DMSO at 70:30 volume ratio, GBL:sulfolane at 70:30 volume ratio, and GBL:sulfolane:AcOH at 70:27.5:2.5 volume ratio, respectively.

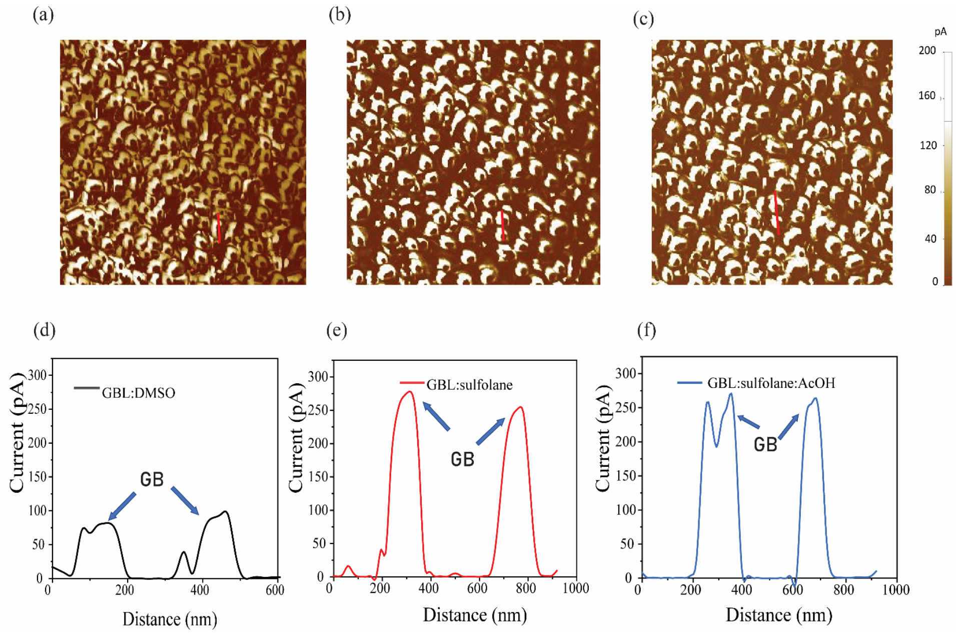

Figure S4: The conductive-AFM image of the perovskite films fabricated using different solvent systems: (a) GBL:DMSO at 70:30 volume ratio, (b) GBL:sulfolane at 70:30 volume, and (c) GBL: sulfolane:AcOH at 70:27.5:2.5 volume ratio. (d-f) The current surface along the line indicated in respective c-AFM images, GB denotes the location of grain boundary.

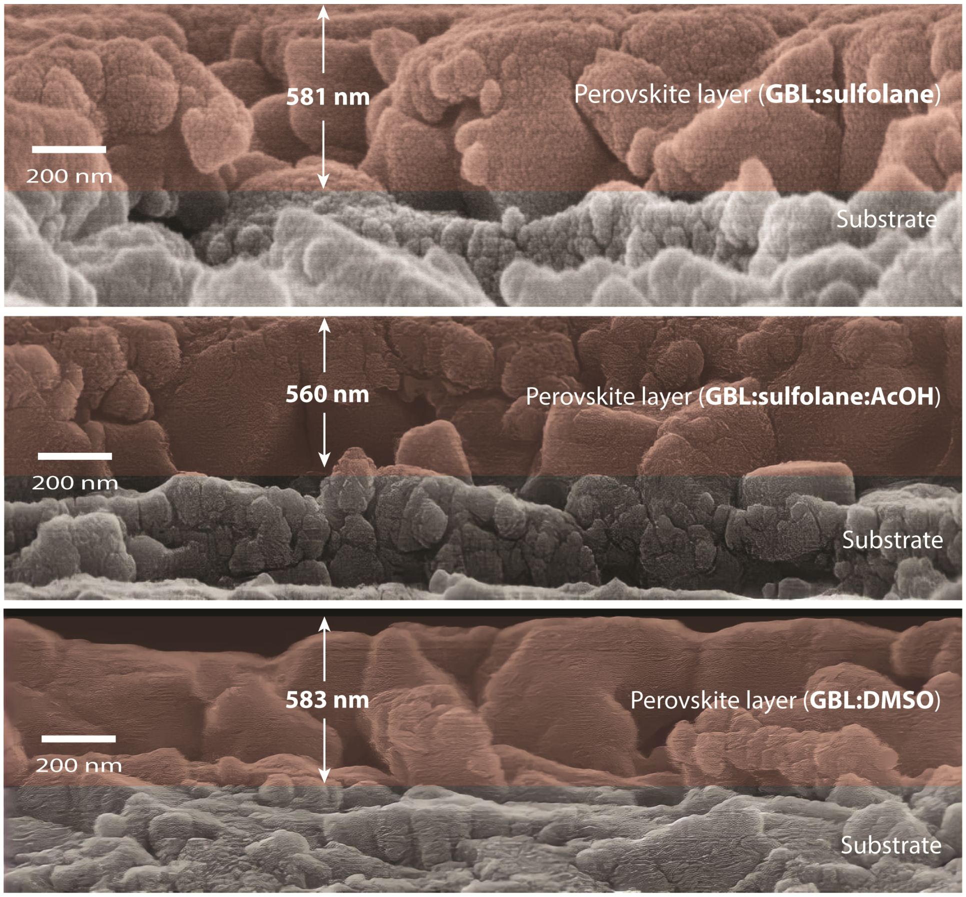

Figure S5: Cross-sectional SEM images of the perovskite films prepared using different solvents.

Analysis of time-resolved photoluminescence

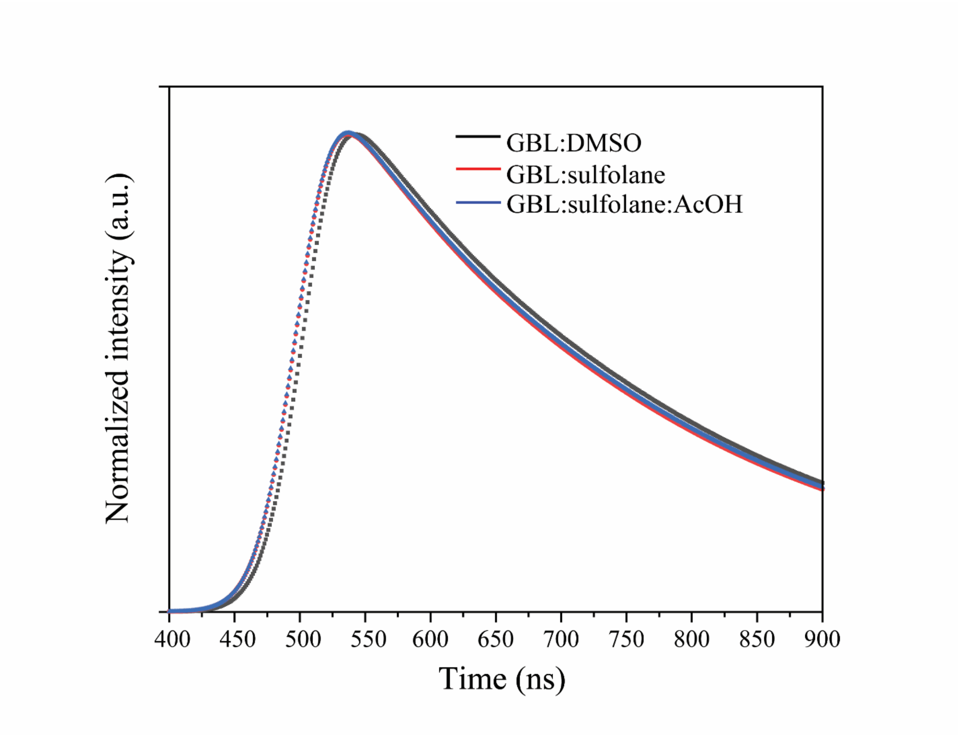

Figure S6: Time-resolved photoluminescence (TRPL) measurement for perovskite films with each solvent system.

The time-resolved photoluminescence intensity was measured using the excitation by using pulse p. The sum of the intensity detected after each excitation pulse p can be accumulated according to equation 3.[1]

$$[H]\mathrm{I} = \sum{}_{p} I_p(t) \tag{3}$$

The intensity decay profile can be fitted using multi-exponential decay model:

$$[H]\mathrm{I(t)} = \sum_i \mathrm{A_i e^{-t_{\tau_i}}} \tag{4}$$

Normalized pre-exponential amplitude can be denoted α(see equation below) and is useful if the actual concentration of each fluorescing component is required. They are easily deduced

from the pre-exponential "A" as shown in equation 5.[1]

$$[H]\alpha_i = \frac{A_i}{\sum_i A_i} \tag{5}$$

where α_i and τ_i are amplitude and lifetime of component i, respectively. Equation 4 can be written as:

$$\mathrm{I}_{\mathrm{TRPL}} = \sum_i \alpha_i e^{-t_{\tau_i}} \tag{6}$$

A bi-exponential (n=2) fit is normally applied for the perovskite material, where the two main components are assigned to radiative recombination related to the electron-hole recombination of the intrinsic electronic structure inside the grains (longer time τ_1) and the recombination process at grain boundaries with the trap states (shorter lifetime τ_2). The fractional intensity of each exponential component (α) usually accounts for the contribution of each process, whereas the lifetime (τ_i)represents the rate of the process.[2] For bi-exponential decay, the average decay time (τ_{AV}) can be calculate using the following equation:

$$[H]\tau_{AV} = \frac{\alpha_1\tau_1^2 + \alpha_2\tau_2^2}{\alpha_1\tau_1 + \alpha_2\tau_2} \tag{7}$$

Table S2: Life time components from the time-resolved photoluminescence measurement of perovskite film with different solvent systems using the analysis of bi-exponential analysis fitting [a].

Solvent system	τ_i(ns)	α_1^b	τ_2(ns)	α_2	τ_{AV}^c(ns)
GBL:DMSO	6.92	0.9662	18.3	0.0337	7.88
GBL:Sulfolane	6.22	0.7259	11.5	0.2741	8.41
GBL:SUlfolane:AcOH	6.58	0.8262	13.3	0.1738	8.60

[a] I(t)=$\alpha_1 e^{-t_{\tau_i}} + \alpha_2 e^{-t_{\tau_i}}$
[b] $\alpha_i = \frac{A_i}{A_1+A_2}$
[c] $T_{av} = \frac{\alpha_1\tau_1^2+\alpha_2\tau_2^2}{\alpha_1\tau_1+\alpha_2\tau_2}$

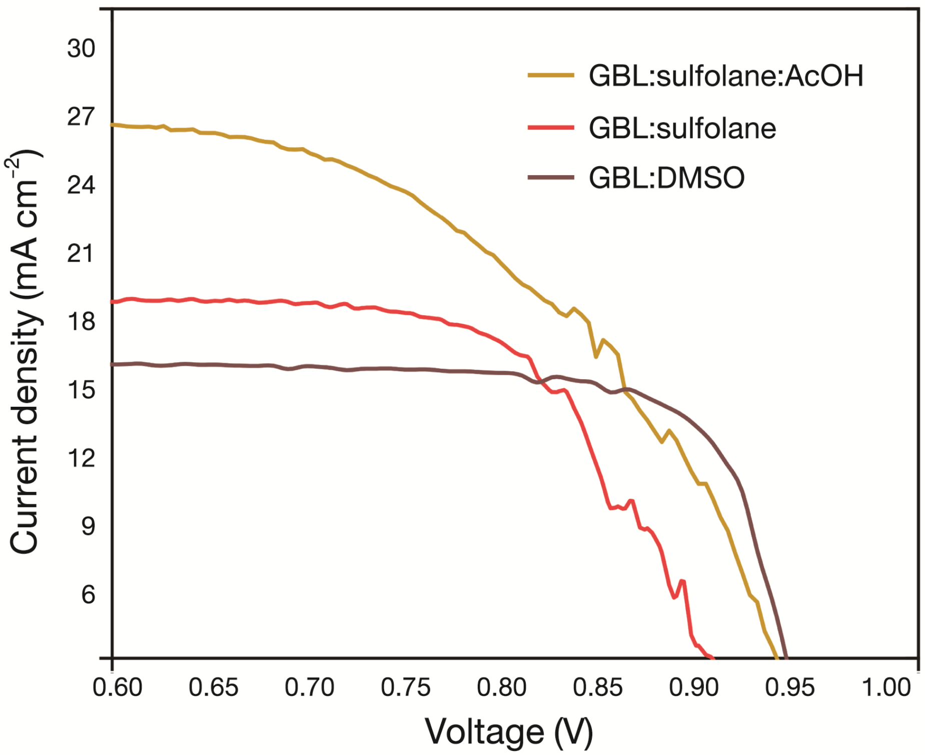

Figure S7: Representative current density (J) - voltage (V) profiles for pervoskite layers prepared using different solvent systems.

References

(1) Sillen, A.; Engelborghs, Y. The Correct Use of "Average" Fluorescence Parameters. *Photochemistry and Photobiology* **1998**, *67*, 475–486.

(2) Teng, P.; Han, X.; Li, J.; Xu, Y.; Kang, L.; Wang, Y.; Yang, Y.; Yu, T. Elegant Face-Down Liquid-Space-Restricted Deposition of $CsPbBr_3$ Films for Efficient Carbon-Based All-Inorganic Planar Perovskite Solar Cells. *ACS Applied Materials & Interfaces* **2018**, *10*, 9541–9546.
